# Closing the AI generalisation gap by adjusting for dermatology condition distribution differences across clinical settings

**DOI:** 10.1016/j.ebiom.2025.105766

**Published:** 2025-06-02

**Authors:** Rajeev V. Rikhye, Aaron Loh, Grace Eunhae Hong, Preeti Singh, Margaret Ann Smith, Vijaytha Muralidharan, Doris Wong, Rory Sayres, Ayush Jain, Michelle Phung, Nicolas Betancourt, Bradley Fong, Rachna Sahasrabudhe, Khoban Nasim, Alec Eschholz, Basil Mustafa, Jan Freyberg, Terry Spitz, Yossi Matias, Greg S. Corrado, Katherine Chou, Dale R. Webster, Peggy Bui, Yuan Liu, Yun Liu, Justin Ko, Steven Lin

**Affiliations:** a Google Research, Mountain View, CA, USA; b Stanford University School of Medicine, Stanford, CA, USA

**Keywords:** Artificial intelligence, Generalisation, Dermatology

## Abstract

**Background:**

Generalisation of artificial intelligence (AI) models to a new setting is challenging. In this study, we seek to understand the robustness of a dermatology (AI) model and whether it generalises from telemedicine cases to a new setting including both patient-submitted photographs (“PAT”) and clinician-taken photographs in-clinic (“CLIN”).

**Methods:**

A retrospective cohort study involving 2500 cases previously unseen by the AI model, including both PAT and CLIN cases, from 22 clinics in the San Francisco Bay Area, spanning November 2015 to January 2021. The primary outcome measure for the AI model and dermatologists was the top-3 accuracy, defined as whether their top 3 differential diagnoses contained the top reference diagnosis from a panel of dermatologists per case.

**Findings:**

The AI performed similarly between PAT and CLIN images (74% top-3 accuracy in CLIN vs. 71% in PAT), however, dermatologists were more accurate in PAT images (79% in CLIN vs. 87% in PAT). We demonstrate that demographic factors were not associated with AI or dermatologist errors; instead several categories of conditions were associated with AI model errors (p < 0.05). Resampling CLIN and PAT to match skin condition distributions to the AI development dataset reduced the observed differences (AI: 84% CLIN vs. 79% PAT; dermatologists: 77% CLIN vs. 89% PAT). We demonstrate a series of steps to close the generalisation gap, requiring progressively more information about the new dataset, ranging from the condition distribution to additional training data for rarer conditions. When using additional training data and testing on the dataset without resampling to match AI development, we observed comparable performance from end-to-end AI model fine tuning (85% in CLIN vs. 83% in PAT) vs. fine tuning solely the classification layer on top of a frozen embedding model (86% in CLIN vs. 84% in PAT).

**Interpretation:**

AI algorithms can be efficiently adapted to new settings without additional training data by recalibrating the existing model, or with targeted data acquisition for rarer conditions and retraining just the final layer.

**Funding:**

10.13039/100006785Google.

**Research in context**

**Evidence before this study**

We searched PubMed and Google Scholar for articles on AI models in dermatology up until Sep 1, 2024. Key search terms included ‘dermatology’ AND (‘machine learning’ OR ‘artificial intelligence’) AND (‘patient’ OR ‘consumer’). Most studies focus on predicting malignancies, and many on dermatoscope images. One study examined generalisation of AI models trained on clinical images to images from a melanoma internet forum and attributed performance differences to image quality and rarer conditions. Additionally, studies have indicated that clinician-taken photographs can be of higher quality than those taken and submitted by patients. However, little was known about the robustness of AI models to different photograph types while controlling for potential confounders such as patient/user population, nor was there insight into the reasons for any differences observed.

**Added value of this study**

We find that a dermatology image AI model, trained primarily on clinician-taken photographs, generalised well to both clinician-taken and patient-taken photographs from a different source. However, in our study, dermatologists demonstrated higher diagnostic accuracy on patient-taken photographs than clinician taken photos. Our analysis suggests that this difference is attributable to differences in skin condition distribution between patient and clinician taken (submitted) photographs. Finally, we introduce data-efficient methods of improving generalisation performance, ranging from using just the expected condition prevalence to calibrate the model outputs, to fine-tuning the classification layers using examples of conditions that are infrequent (i.e., out-of-distribution) in the training dataset.

**Implications of all the available evidence**

When the AI model is trained on a sufficiently diverse dataset including data from multiple sources and environments, the photographer (clinician or patient) and corresponding differences in devices, environment, and training may matter less. However, the conditions distribution differences may still affect performance. Efficient ways to close a generalisation gap include calibrating the AI model’s outputs using an estimated condition distribution, and fine-tuning the classification layers of the AI model on additional rarer conditions. The latter approach can be particularly convenient if leveraging embedding models that provide a pre-trained model up until the penultimate layer before the classification layer.

## Introduction

Since the COVID-19 pandemic, skin conditions have grown to the fifth most common concern in telehealth in the United States.^1^ More healthcare providers are assessing dermatological conditions from digital photographs–such as those captured during clinical examination by a non-specialist or taken by the patient remotely. In parallel, several artificial intelligence (AI) algorithms have been developed to help interpret images of skin conditions.^2^ While these algorithms achieve similar or better performance than clinicians in experimental settings,^3^, ^4^, ^5^ factors such as differences in case ambiguity,^3^^,^^6^^,^^7^ skin tone distribution^8^ or dermatological condition differences between the training and evaluation set can cause differences in AI algorithm accuracy and reduced usefulness in real-world settings. However, little is known about the robustness of these algorithms to previously unseen data sources,^9^ such as a different institution or data acquisition device.

In this retrospective study, we evaluated the performance of a deep learning-based AI model for skin condition classification on a dataset from Stanford Health Care, not previously seen by the AI. Unlike both the training dataset and data used in most research studies, this dataset was enriched for patient-taken images (PAT, comprising approximately 25% of this dataset) instead of clinician-taken (CLIN, approximately 75%) images. Compared to the training set, there were substantial differences in the condition distribution, which we found contributed to lower AI accuracy for this dataset. We demonstrate a series of steps for refining the algorithm to help improve AI generalisation to new environments or dataset shifts.

## Methods

### Study setup and dataset

This retrospective cohort study included 2500 de-identified cases from Stanford Health Care’s eConsult system between November 2015 and January 2021, including cases from 22 unique clinics across the San Francisco Bay Area. Based on census data, the distribution of ethnicities in our samples is representative of the Bay Area population. Out of the 22 clinics, 89% were primary care facilities (family medicine, urgent care, etc.) and the rest were internal medicine facilities. We observed no differences in age, ethnicity, condition category or case difficulty between the clinics, and thus aggregated cases over the clinics. We noticed a change in the fraction of CLIN cases relative to PAT cases over time, as noted in a prior study.^10^ In terms of imaging technology (i.e., the make of the camera), we only had EXIF (metadata) information from roughly 10% of the data. Within that small slice, we found no appreciable difference between camera models. As such, we have not stratified our analysis temporally or by device.

To improve the relative representation of rarer conditions while maintaining a reasonable number of cases for de-identification and analysis, upstream of the de-identification workflow, we sub-sampled 20% of cases with eczema (ICD10: L20-L30); 50% of cases with seborrhoeic keratoses or irritated seborrhoeic keratosis (ICD10: L82) and 50% of cases with rashes (ICD10: R21). Unless otherwise indicated, all results presented are adjusted for this sampling. The final sample size was determined qualitatively with the goal of ensuring sufficient numbers of CLIN and PAT cases for analysis of differences, while ensuring available personnel-time for de-identification. This determination was qualitative because it was not possible to confirm if a given case was CLIN or PAT prior to manual review of the case and images.

### Case de-identification

Case information, including images of skin conditions and clinical metadata, were stripped of all protected health information and subject identifiers. For each case, a Stanford research team member reviewed all images and clinical metadata. Any image containing identifying information (e.g., full face, unique traits such as a birthmark, piercing, or tattoo, etc.) was cropped or removed.

**Fig. 1:**
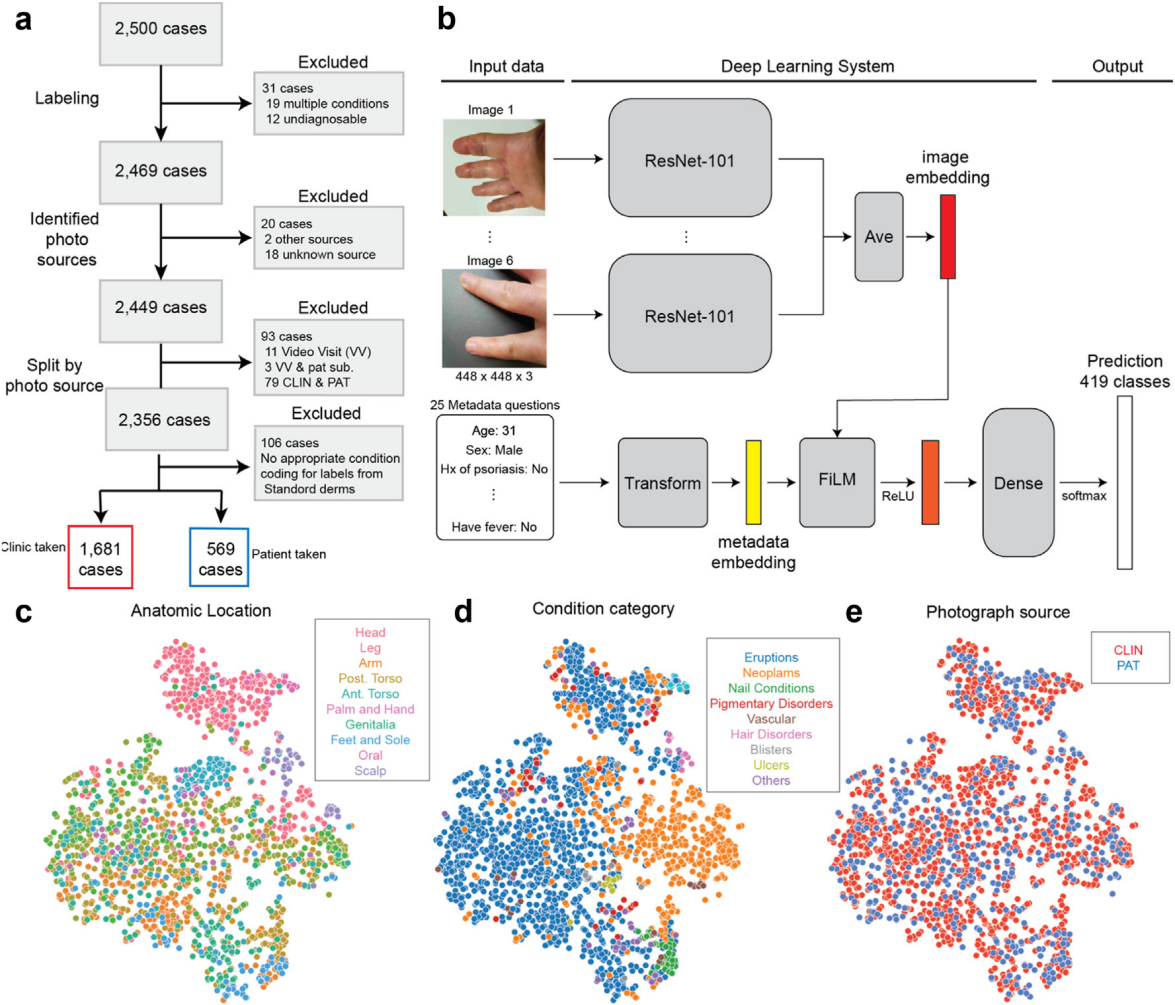
Study flow, AI model architecture, and AI embedding visualisation. a: Case selection procedure. Characteristics of the PAT and CLIN datasets are presented in Table 1, Table 2. b: Model architecture. c–e: Two dimensional projections of the combined image-metadata embedding coloured by anatomic location (c), condition category (d) and photograph source (e). For a projection visualization comparing before vs. after retraining, see Supplementary Fig. S1. Saliency map examples for sample cases are shown in Supplementary Fig. S2 and a more granular breakdown of the embedding projections (panels c and d) is presented in Supplementary Fig. S3. Mappings of conditions to categories are provided in Supplementary Table S6.

Age, sex, clinician-estimated Fitzpatrick skin type (eFST), photo source and other diagnostic metadata questions were extracted from the health record per case. If absent in the record, FST were visually assessed retrospectively. Cases with either “unknown” or “other” photo source were excluded (20/2500). A small fraction of cases (79/2500) had both CLIN and PAT images and were excluded due to the ambiguity in the case-level PAT/CLIN classification and small numbers precluding robust analysis. CLIN cases were captured using the Epic HAIKU application on mobile phones. Cases containing video visit screenshots (14/2500) were also excluded from this study to retain only photographs from a mobile phone. The remaining cases were stratified into CLIN and PAT (Fig. 1a). Photographer information for CLIN cases (e.g. physician or nurse) was unavailable, and metadata such as the smartphone used was not available for most photos. More details on the differences between CLIN and PAT are in Tables 1 and 2 and Rikhye et al.^10^

**Table 1:** Descriptive statistics of demographic variables in this dataset.

	CLIN	PAT
**Sex (n, %)**		
Female	807 (48.0%)	320 (59.3%)
Male	670 (39.9%)	219 (40.6%)
**Age (n, %)**		
≥60	409 (24.3%)	125 (23.2%)
50-59	210 (12.5%)	79 (14.7%)
40-49	218 (13.0%)	97 (18.0%)
30-39	355 (21.1%)	145 (26.9%)
<30	285 (17.0%)	93 (17.3%)
**eFST (n, % excluding unknown)**		
Unk.	289	247
V/VI	53 (4.5%)	10 (3.42%)
III/IV	548 (46.1%)	145 (49.7%)
I/II	587 (49.4%)	137 (46.9%)

Information were extracted from the electronic health record.

**Table 2:** Descriptive statistics of clinical variables in this dataset.

	CLIN	PAT
**Condition category (n, %)**		
Infections	240 (14.3%)	63 (11.7%)
Contact dermatitis	271 (23.2%)	113 (21.0%)
Inflammatory	280 (24.0%)	112 (20.8%)
Other eruptions	183 (15.7%)	65 (12.1%)
Neoplasms	334 (28.6%)	97 (18.0%)
Pigmentary disorder	34 (2.9%)	17 (3.0%)
Vascular	29 (2.5%)	12 (2.2%)
Blisters and ulcers	16 (1.4%)	5 (0.9%)
Hair disorders	10 (0.9%)	8 (1.5%)
Nail disorders	30 (2.6%)	17 (3.2%)
Others	49 (4.2%)	30 (5.6%)
**Anatomic location (n, %)**		
Scalp	32 (2.7%)	18 (3.34%)
Face and neck	346 (29.6%)	132 (24.5%)
Mouth and tongue	15 (1.3%)	4 (0.7%)
Arm	232 (19.9%)	73 (13.5%)
Hand and palm	87 (7.4%)	28 (5.2%)
Anterior Torso	191 (16.4%)	72 (13.4%)
Posterior Torso	103 (8.8%)	37 (6.9%)
Genitalia	61 (5.2%)	22 (4.1%)
Buttocks	31 (2.7%)	5 (0.9%)
Leg	254 (21.7%)	100 (18.6%)
Dorsal foot	40 (3.4%)	26 (4.8%)
Unspecified	85 (7.3%)	22 (4.1%)
**Inter-rater agreements**		
Disagreement (0/3)	459 (39.2%)	145 (26.9%)
Intermediate (2/3)	342 (29.3%)	145 (26.9%)
Unanimous (3/3)	646 (55.3%)	249 (46.2%)

Regarding data missingness, demographic data (such as, age, sex) presented in this manuscript were available for most patients. This did not impact the AI model evaluation (which does not rely on these data being available; the age/sex input as well as many of the structured medical history fields can be “not available”. Thus imputation (which can create its own set of biases) was not done. We reason that the basic demographic variables could potentially be missing completely at random (such as data entry issues), but for the missingness at each step of the study flowchart (Fig. 1a), these are likely a complex mix of missing at random and missing not at random. For example, each step’s exclusions will have its own correlations, such as the 19 “multiple conditions” potentially being more complex cases, and the 11 video visits correlated with healthcare access or technology literacy. However, except for the 79 CLIN/PAT mix of images where patients performed both an in-person visit and submitted photos online (and hence cannot be cleanly categorised into the CLIN vs. PAT buckets), each of these exclusions are relatively rare at 20 or fewer instances. Therefore, we expect that the impact of these exclusions (which ensure that the analysis is as clean and easy to interpret as possible) is minimal.

### Ethics

This study was approved by the Institutional Review Board of Stanford University Medical Centre (Protocol # 59290) and conducted according to the Declaration of Helsinki. Patients provided written consent for their electronic health record (EHR) data, including photos, to be used secondarily in research projects in a de-identified fashion in line with Stanford University’s general treatment authorisation procedure. Case information, including images of skin conditions and clinical metadata, were stripped of all protected health information and subject identifiers. For each case, a Stanford research team member reviewed all images and clinical metadata. Any image containing identifying information (e.g., full face, unique traits such as a birthmark, piercing, or tattoo, etc.) was cropped or removed.

### Reference diagnosis

In addition to the Stanford dermatologist’s assessment, three US board-certified dermatologists independently provided a differential diagnosis and confidence score per diagnosis per case.^3^ Each case included up to six photographs, demographic information and structured medical history. First, each of the three panel dermatologists assigned to a case provided a differential diagnosis and accompanying confidence values in the range [1–5] for each of the diagnoses. Next, each diagnosis was mapped to a list of 419 conditions. If duplicates occurred (i.e., multiple diagnoses were mapped to the same condition), the highest confidence was retained. Diagnoses that could not be mapped to the 419 conditions were labelled as “not categorised” and were subsequently dropped. Next, the conditions were ranked into a differential diagnosis based on their relative confidence values. Each condition in the differential diagnosis was then assigned a weight, which was the inverse of their rank. The differential diagnoses from multiple dermatologists were then combined using a ‘collective intelligence’ approach; and more details can be found in the Supplementary methods in Liu et al.^3^

Cases assessed by the dermatologists to have multiple conditions (n = 19), undiagnosable conditions (n = 12) due to image quality issues, minimal visible pathology, limited field-of-view, or conditions that were not supported were excluded. Based on inter-rater agreement, cases were then subclassified as unambiguous (3/3 dermatologists in the panel agreed), intermediate (2/3 agreed) or ambiguous (all disagreed).

### Skin condition classification AI

The skin condition AI model used in this study takes as input up to 6 photographs and a list of 25 structured metadata questions (such as medical history information) and returns a prediction over 419 possible skin conditions (Fig. 1b). The model resembles Liu et al.^3^ with a few key differences. First, this model uses wide ResNet-101x3 feature extractors that were pretrained on web scale images using contrastive learning.^11^ Briefly, each image had a paired text description of that image. Embeddings were computed for both image and text and a CLIP-style loss was used to ensure that similarity between similar conditions was maximised. For more detail, see Zhang et al.^12^

For each patient, we selected 6 images, sampling at random if cases had more than 6 images. In prior work,^3^ most (75%) of cases had 6 or fewer images; in this current cohort we found that the number of images typically was between 3 and 4 per case,^10^ and less than 5% had more than 6 images per case for both CLIN (red dots) and PAT (blue dots), with patients submitting more photographs than clinicians. We also found that the number of images per case varied with the condition category. Given the small counts of cases that had more than 6 images, this was not changed for validation on this external dataset.

For each image, an image embedding was computed and embeddings across images were combined via an average-reduce. The metadata comprised 25 different categorical variables including patient history (e.g. history of psoriasis, etc.), signs (e.g. redness, swelling, etc.), symptoms (e.g. itching, fever, etc.) and demographic factors (e.g. sex, age and eFST). All categorical questions were one-hot encoded and concatenated with age before being projected into a metadata embedding using feature-wise linear modulation (FiLM). The AI was trained using 41,290 cases (194,461 images) covering 204 unique primary conditions.

To visualise the resulting embedding in two dimensions (Fig. 1c), we used UMAP (Uniform Manifold Approximation and Projection) to perform non-linear dimensionality reduction. The points, which represent each case, were then coloured by either skin condition category, anatomic location or photo source (CLIN, PAT).

### Technical training details

The metadata embedding and image embedding were combined using feature-wise linear modulation (FiLM) which is defined as follows:

$$FiLM\left(E_{image+metadata}\right)=\beta \left(E_{metadata}\right)+\alpha \left(E_{metadata}\right)\circ E_{image}$$

where, $\alpha \left(E_{metadata}\right)$ and $\beta \left(E_{metadata}\right)$ are learned projections of the metadata embeddings. Therefore, FiLM applies an affine transformation to the image embedding ($E_{image}$) by scaling it by $\alpha \left(E_{metadata}\right)$ and shifting it by $\beta \left(E_{metadata}\right)$. This in turn results in image embedding features that are modulated by features in the metadata.

The focal loss^13^ was used to account for class imbalance:

$$FL\left(p_{t}\right)=-\alpha {\left(1\;-\;p_{t}\right)}^{\gamma }\;\log\left(p_{t}\right)$$

where the parameters α and γ are selected via hyperparameter tuning to maximise top-3 accuracy. Focal loss, which is a scaled form of cross entropy, was selected given the class imbalance in the development set, allowing the model to learn more from hard-to-classify examples. A constant learning rate schedule was used along with the ADAM (adaptive moment estimation) optimiser and the number of training steps was also selected via hyperparameter tuning. To make the model robust to missing metadata we used dropout where we replaced metadata fields with “unknown” values at random with a 25% probability.

### Variable-k accuracy

Increasing the number of conditions output by the model improves the accuracy, since there is a higher likelihood of including the reference diagnosis. However, this approach also leads to a higher false positive rate by outputting less relevant conditions. We developed a method^14^ that allowed us to dynamically select the *k* diagnoses on a case-by-case basis while maintaining the overall sensitivity at 95%. Thus in addition to top-3 accuracy, where the top-1 reference diagnosis has to be within the top-3 predictions, we also report top variable-k accuracy. Specifically, the model can customise the number of predictions per case, with smaller k for confident predictions and larger k for uncertain cases. Using the development set, we computed a score threshold that maximised the sensitivity for high risk conditions (e.g. certain skin cancers) and minimised the false positive rate for an output between 3 and 7 predictions. During inference, we added prediction scores for the top-k predictions until the threshold was reached. Accuracy against the top-1 ground truth was then computed.

### Score recalibration

The output of the model is a probability distribution over labels (419) and is said to be well calibrated if and only if $p\left(c_{j}\;|\;p^{c_{j}}\right)=p^{c_{j}}$, where $p^{c_{j}}$ is the probability estimate for class j. In other words, if a number of predictions with probability estimate 0.8 are made, then the predictions should be correct in 80% of the cases. To correct for distribution differences between the development (DEV) and Stanford data sets, we employed a multiclass version of Platt scaling.^15^ Platt scaling fits a sigmoid function (z) to the scores (z) obtained by the model on the calibration set

$$\sigma \left(z^{k}\right)=\exp\left(z^{k}/T\right)/\sum_{j=1..K}\exp\left(z^{j}/T\right)$$

Where T is the temperature parameter and k is the condition category. The parameter T is learned by performing one-vs-rest classification for each of the 11 condition categories on 20% of Stanford cases (via stratified sampling). This calibration set was identified by stratified sampling.

### Statistics

We evaluated the AI using the top-3 accuracy, defined as whether the top-1 reference diagnosis (from the dermatologist panel) is within the top-3 predictions. For example, if the reference diagnoses for a case are: {A, B, C}, then the top diagnosis is “A”. If the AI’s predicted conditions for that case are {D, E, A}, its top-3 accuracy for this case is 1.0, because diagnosis “A” is in its first 3 predictions. In addition, we also report top-k accuracy where *k* varies per case. Specifically, the AI can customise the number of predictions per case, with smaller *k* for confident predictions and larger *k* for uncertain cases. Subgroup analyses focused on demographic factors: sex, age, eFST, to understand potential fairness/equity issues.

**Fig. 2:**
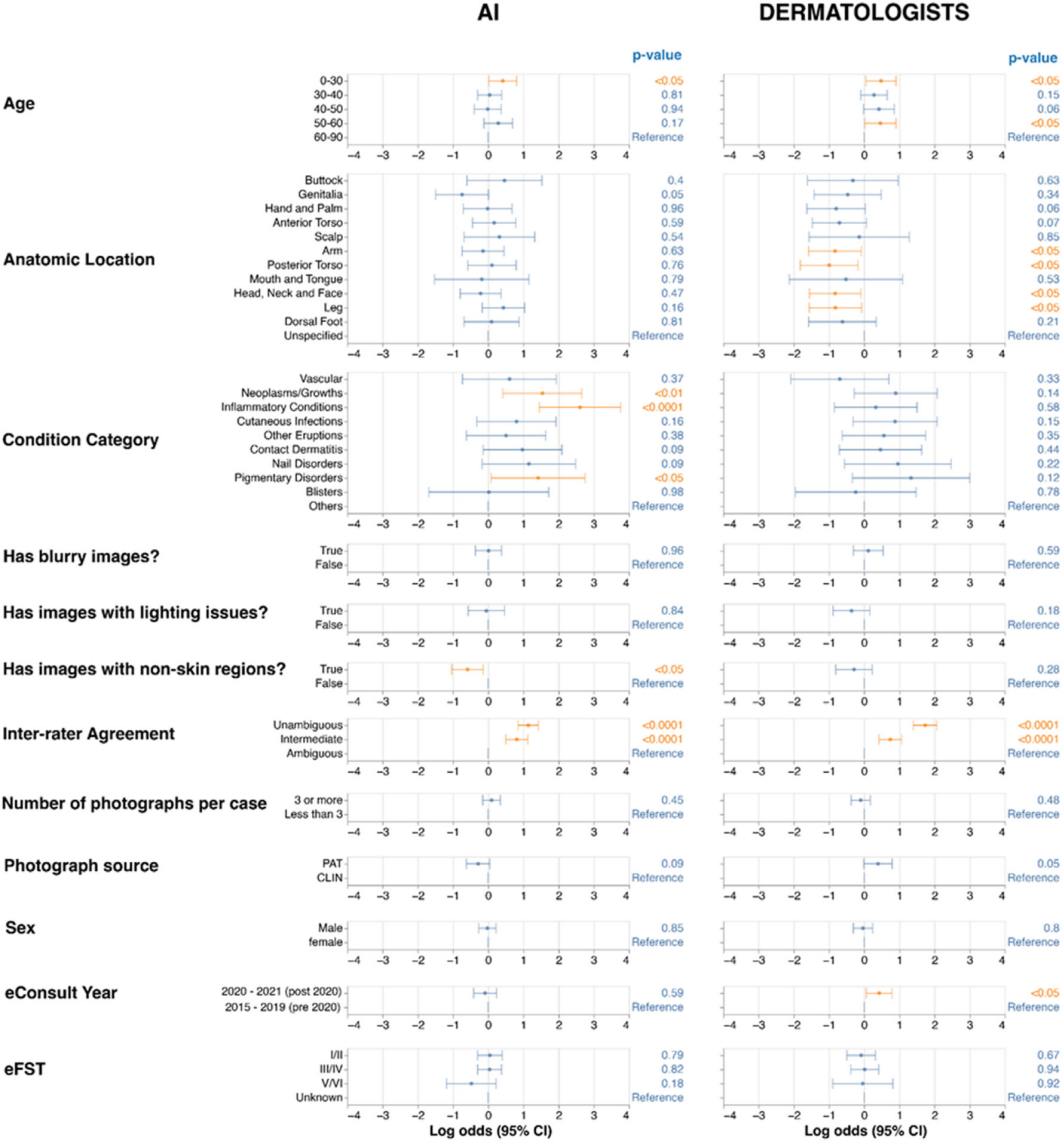
Association between demographic, clinical and image quality factors and accuracy. This analysis includes the full dataset of PAT and CLIN (n = 2250). Red coloured points are statistically significant at an alpha level of 0.05 after Bonferroni correction per variable (e.g., 3 age groups). Significance was assessed using the Mann–Whitney U-test. The asterisks indicate that the point is outside the range of the graph, and indicated separately to aid visualisation of the remaining points. Log odds were used instead of odds ratio to ensure left-right visual symmetry. Both the AI (left) and dermatologists (right) are less accurate in more difficult cases with inter-rater disagreement within the dermatologist panel. For the AI, several condition categories and images with substantial amounts of non-skin areas were also significantly associated with errors. Tabular data for univariable and multivariable analyses are presented in Supplementary Table S7.

To understand what factors were associated with AI model and dermatologist errors and could confound interpretation of the raw accuracy results, we applied multivariable logistic regression independently for^1^ the AI model and^2^ dermatologists. The independent variables were demographic factors, case ambiguity, condition categories, anatomic location of the lesion, whether the case was pre-COVID (defined as before 2020), and various image quality factors (Fig. 2). The dependent variables for the 2 multivariable models were respectively the AI model and dermatologists’ top-3 accuracy relative to the ground truth. For example, if for a given case the ground truth was in the top-3 predictions made by the AI model, we labelled the case as correct and the dependent variable was “1”. Conversely, if the ground truth was not in the top-3 prediction, the dependent variable was “0”. The independent variables were assigned based on the case. Thus the effects for each independent variable captures whether that variable was associated with the AI model (or dermatologist) correctness; a positive log odds indicates that variable is positively associated with the AI model (or dermatologist) being correct, whereas a negative log odds indicates that variable is associated with the AI model (or dermatologist) being wrong.

### AI refinement approaches

To improve performance on a new dataset, we present several approaches that vary based on the amount of the information required and the computation needed. The first, score recalibration, requires information about the expected condition distribution in the new dataset, but does not require retraining the model. The second, condition distribution matching, uses information about the expected condition to resample the development dataset and retrain or fine-tune the model. The third approach is to specifically obtain rarer conditions (from the new dataset) that are lacking in the development dataset, and augment the original training or fine-tuning. A final approach involved training just the final layer of the AI model instead of the full model, which is substantially faster.

### Matching development (DEV) data to stanford condition distribution

In order to match the DEV set to the Stanford condition distribution, we first compute the probability distribution of the Stanford dataset (CLIN and PAT combined) over the 11 condition categories. Next, we use the Metropolis–Hastings algorithm to generate samples from the DEV set with a fixed random seed. The AI was either retrained or fine-tuned using this DEV dataset.

### Augmenting training data with stanford examples

In order to augment the DEV set with examples from Stanford, we first identified conditions in the Stanford dataset that were less common in the DEV set by comparing the two histograms. Next, we sampled cases from the less common conditions with probability α=0.7 and cases from more common conditions with probability 1−α. These cases were then added to the existing training set. In this way, the model was trained on both Stanford conditions and images of those conditions. The sampled cases were drawn from a 20% subset of PAT and CLIN (stratified by condition and demographics), and evaluation was done using the remaining 80%. To ensure comparability across these experiments (recalibration, training data changes, tuning), the evaluations used the same 80% set.

### Role of funders

Google funded this work and Google employees were involved in study design, data (label) collection, data analysis, interpretation, and writing of the reports.

## Results

We first sought to understand if the AI which was trained primarily (though not exclusively) on clinician taken images (CLIN) could generalise to patient-taken images (PAT), which are, on average, poorer quality than photographs taken by clinicians in prior studies.^16^^,^^17^ The retrospective cohort included 1719 CLIN cases and 628 PAT cases from 22 clinics across the San Francisco Bay Area (Methods); descriptive statistics and dermatology conditions involved are reported in Table 1, Table 2 and prior work.^10^

We measured the top-3 accuracy and surprisingly, we found no statistical difference in the AI’s top-3 accuracy between CLIN and PAT (Table 3a, top-3 accuracies between 71% and 74%, p = 0.214, ANOVA), suggesting that the AI performed similarly on both photo sources. Comparisons with an internal test dataset (i.e., cases from similar sources to that used in training, but where the specific cases were not used for training) are shown in Table 4.

**Table 3:** Comparison between AI and Stanford dermatologist (Derm) accuracy for both CLIN and PAT.

a) Performance on original dataset				
	CLIN (n = 1681 patients)		PAT (n = 569 patients)	
	AI	Stanford Derm	AI	Stanford Derm
Top-3 accuracy	73.6% (71.2, 75.9)	78.7% (76.4, 80.9)	70.7% (66.3, 74.8)	87.3% (83.7, 90.3)
Top-k accuracy	84.6% (82.8, 86.4)	80.5% (78.2, 82.6)	82.5% (78.9, 85.7)	88.7% (85.4, 91.6)

b) Performance after resampling 500 cases to match the development dataset condition distribution				
	Re-sampled CLIN (n = 500 patients)		Re-sampled PAT (n = 500 patients)	
	AI	Stanford Derm	AI	Stanford Derm
Top-3 accuracy	83.6% (78.7, 87.8)	77.3% (71.1, 82.7)	79.1% (72.9, 84.5)	88.9% (83.5, 93.0)
Top-k accuracy	93.0% (89.4, 95.6)	79.5% (73.6, 84.5)	87.8% (82.8, 91.8)	91.9% (86.7, 95.4)

The upper table (indicated by a) represents performance on the entire dataset; the lower table (indicated by b) represents performances on the data after resampling cases to match the development dataset condition distribution. The 95% confidence intervals are shown in parentheses.

**Table 4:** Comparison of top-3 accuracy between the held-out development (DEV) set and Stanford datasets.

	Stanford Data		Internal dataset	
	CLIN (n = 1681)	PAT (n = 569)	DEV (n = 1000)	DEV matched (n = 1000)
AI	73.6% (71.2, 75.9)	70.7% (66.3, 74.8)	82.6% (81.9, 91.6)	78.9% (76.2, 81.9)

DEV-matched refers to DEV cases that were resampled to match the Stanford distribution. All n values refer to the number of patients.

To further understand this observation, we visualised the AI’s embedding by projecting it to two dimensions (Fig. 1c) and found that the embedding clusters by condition category and body location. For example, eruptions on the face are distinct (in embedding space) from those on the torso. Interestingly, we do not notice clustering by photograph source (CLIN vs. PAT), which suggests that the AI extracts similar features regardless of who took the photograph (i.e., clinically trained vs. untrained individuals) and the corresponding differences in background (clinical setting vs. non-clinical).

Interestingly, Stanford dermatologists’ (Derm) diagnoses had higher agreement with the reference diagnosis for PAT than for CLIN (87% vs. 79%, Table 3a). This higher PAT accuracy resulted in a greater difference between Derm and AI performance for PAT (87% Derm vs. 71% AI, difference of 17%) than for Clin (79% Derm vs. 74% AI, difference of 5%). In other words, even though AI performance was similar between the two sets, AI performance relative to dermatologists differed, suggesting that the AI performance was lower after “controlling” for dermatologist performance. Supplementary Table S1 shows the top-3 accuracy stratified by case ambiguity (Methods). As expected, both the AI and Derm have higher accuracy for unambiguous cases (where all 3 dermatologists are in agreement; 82% and 92% for CLIN, 72% and 95% for PAT) than the ‘medium’ cases (with 2 of 3 agreement; 75% and 75% for CLIN, 76% and 87% for PAT) and ambiguous cases (without inter-dermatologist agreement; 63% and 62% for CLIN, 62% and 73% for PAT). For the least ambiguous cases in particular where the reference diagnosis is more certain, the AI performs better on CLIN than PAT (82% and 72% respectively), while Derms have comparable accuracy (>90%).

In terms of the top-k accuracy, where the AI determines if additional predictions should be included in the results (see Supplementary), we found similar performance between the AI and Derm for both CLIN and PAT (Table 3a; 83–85% for the AI vs. 81–89% for Derm). We also observed no differences in the number of conditions output by the AI between CLIN and PAT cases (Supplementary Table S2). This suggests that the AI had identified relevant conditions (e.g. within the top 7), but was not ranking them highly enough (e.g. within the top 3) to achieve comparable top-3 accuracies.

### Demographic and image quality factors with model performance

We next performed multivariable analysis to determine the extent to which differences in demographic (Table 1), clinical (Table 2), and image quality factors^10^ are associated with differences in model performance for CLIN and PAT images (Fig. 2). Demographic factors such as age, sex and eFST were not associated (p > 0.05, Mann–Whitney U-test) with either the AI or the Derm making a correct prediction (see also Supplementary Table S3). Relative to ambiguous cases, unambiguous and intermediate cases were associated (p = 0.00057 and p = 0.00038, respectively, both Mann–Whitney U-test) with correct predictions for both the AI and Derm. This further underscores the finding that case ambiguity or difficulty has an important impact on both AI and Derm accuracy.

Similar to Derm, the AI was fairly robust to image quality differences and only images with significant non-skin areas, such as clothes or background, were associated (p < 0.05, Mann–Whitney U-test) with incorrect AI predictions. The effect was similar in direction for Derms but was not statistically significant (p = 0.28, Mann–Whitney U-test). Interestingly, while the skin condition category was not associated with Derm accuracy, certain categories such as neoplasms, inflammatory conditions and hair conditions were associated with higher AI accuracy. Anatomic location of the skin condition was not associated with AI accuracy, though lesions on genitalia were associated with lower Derm accuracy. Together this suggests that AI accuracy could be impacted by the presence of conditions that the AI was not trained to expect. Interestingly, a trend (albeit non-significant post adjustment) was observed where later years were associated with higher Derm accuracy (the reverse of that seen for the AI), possibly signalling a learning effect by dermatologists getting used to the eConsult system, or a shift towards greater utilisation and easier condition distributions with time.

### Condition distribution differences contribute to lower model performance

**Fig. 3:**
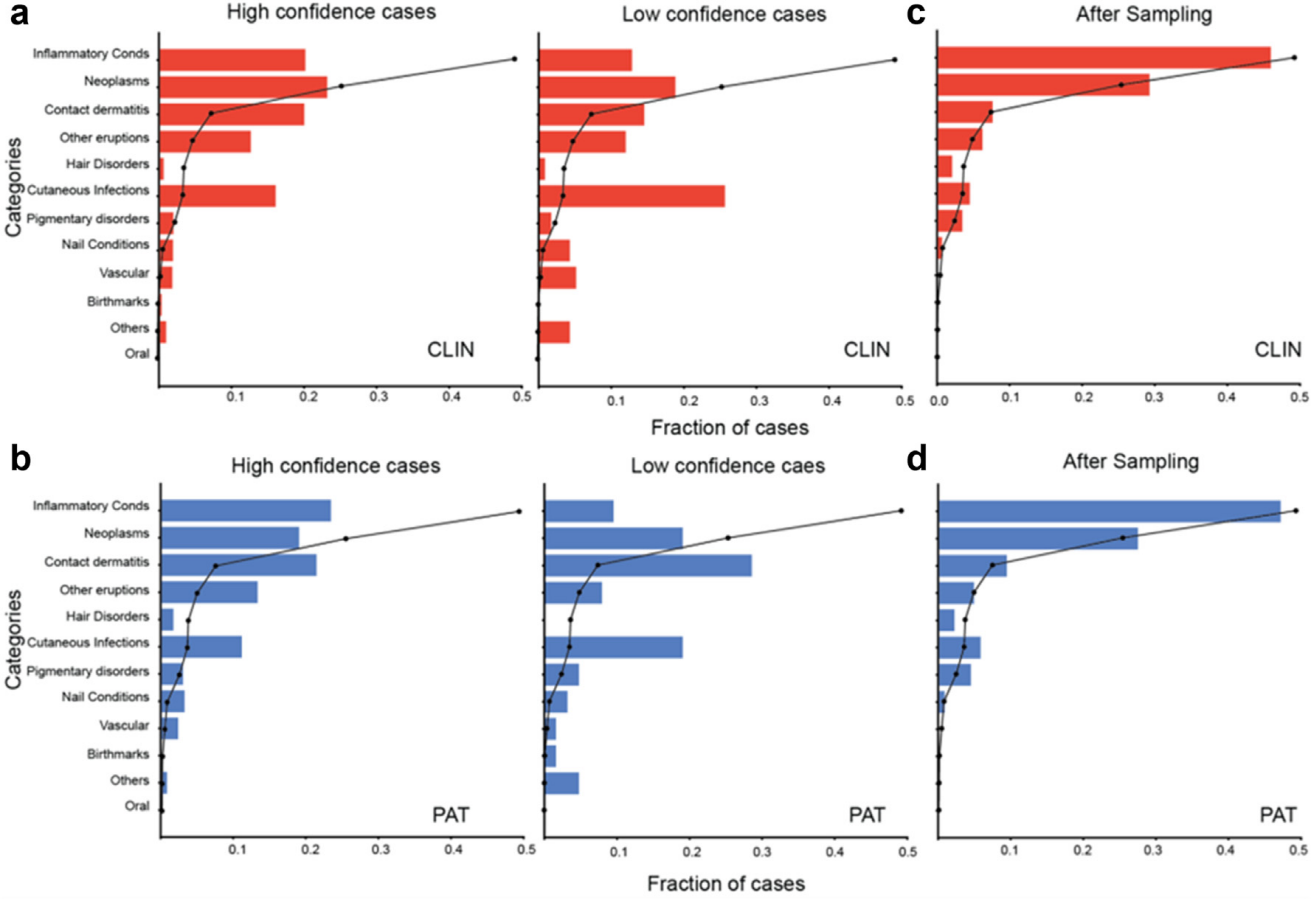
Comparison of condition distributions. a and b: Comparison between CLIN (a, red) and PAT (b, blue) relative to the development set (black) for both high (left) and low (right) confidence cases. c and d: Condition distributions for CLIN and PAT after resampling to match the development set distribution.

As highlighted by the multivariable regression analysis, certain skin conditions categories are significantly associated with AI performance. We hypothesised that differences in condition distributions between the AI development dataset and the Stanford dataset may be responsible for relative AI-Derm differences in performance across PAT and CLIN cases. In particular, inflammatory conditions (such as acne, eczema and psoriasis), neoplasms (melanocytic nevus) and contact dermatitis were much more prevalent in the AI development dataset, while cutaneous infections (such as intertrigo, tinea, herpes zoster) were more prevalent in both Stanford PAT/CLIN subsets. The cases for which the model was more confident (i.e., max classifier score > threshold) had condition distributions that were more similar to the AI development dataset compared to lower confidence cases. Similarly, the long tail of conditions (in the AI development dataset) had a higher incidence in the low confidence cases (Fig. 3a and b).

To explore this notion further, we sub-sampled cases in both CLIN and PAT datasets to match the AI development set distribution (Fig. 3c and d). Evaluating the AI on these cases resulted in an 10.0% absolute improvement in accuracy for CLIN and 8.4% for PAT (Table 3b). Altogether, these results suggest that dermatological AI model generalisation to new datasets is hindered by an unexpected condition distribution. In particular, the closer the distribution is to the training set, the better will be the model performance.

### Fine tuning strategies for closing this generalisation gap

**Fig. 4:**
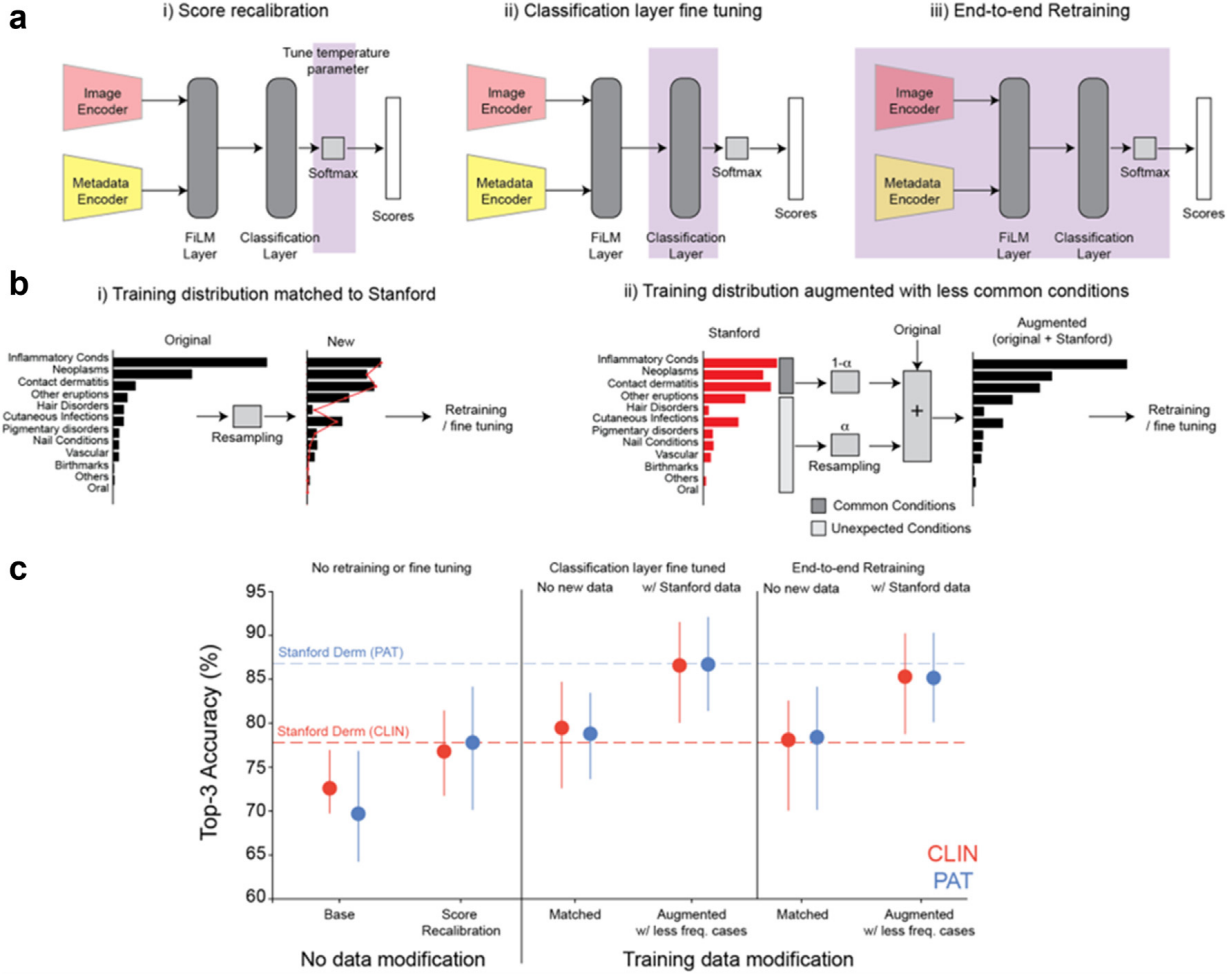
Fine tuning closes the gap between AI and stanford dermatologists. a: Schematic illustrating the various model changes made. b: Schematic illustrating the two different modifications applied to the training data. In (i) the training distribution is resampled to match the Stanford distribution. In (ii) the training distribution is augmented with case examples from Stanford of less frequent conditions. c: Summary plot showing changes in top-3 accuracy after applying different fine tuning methods (n = 1340 for CLIN and n = 400 for PAT). Error bars represent 95% confidence intervals.

We next explored four different methods for closing the generalisation gap, using progressively more information about the CLIN and PAT datasets, and more computational resources (Fig. 4 and Supplementary Tables S6–S8).

First, we developed a multiclass version of Platt scaling^15^ to recalibrate the scores to the distribution expected in the Stanford dataset (Fig. 3a). This recalibration helps the AI model “expect” a different condition distribution after it was already trained. This resulted in a 4% and 8% absolute improvement in top-3 accuracy for CLIN and PAT respectively (Fig. 3c). Despite this, a performance gap remained between AI and Derm.

Second, similar in spirit to the test set condition distribution resampling experiment in Fig. 3, we resampled the training distribution to match the Stanford distribution and retrained the entire model end-to-end with this new development dataset. Since this resampling was done in a condition and demographics-aware manner, we found no significant difference in the demographic distribution of the resampled evaluation dataset (see Methods and Supplementary Table S4). Score recalibration was performed on the re-trained model as mentioned above. This further (albeit to a smaller extent) improved the model performance, underscoring the importance of condition distribution matching in AI generalisation. It is important to note that in both these methods, the model is not exposed to patient images from Stanford and any improvements are solely through using information about the different condition distribution.

Third, we developed a condition-aware sampling method that augmented the existing development set with “unexpected” condition cases (i.e., those less common in development) from the Stanford dataset (Fig. 3b and Supplementary). Unlike the previous methods, this augments the training set with images from Stanford. We retrained the entire model and found a ∼13% improvement in top-3 accuracy for both CLIN and PAT sets. This condition-aware sampling method was better than the common approach of augmenting the training set by randomly selecting 20% of the data (Table 5). This method of fine tuning did not change the subgroup performance of the model across demographic factors (Supplementary Table S5).

**Table 5:** Comparison between AI and dermatologist accuracy for both CLIN and PAT on a hold out set after two different fine tuning strategies were used to re-train the model.

**Techniques applied**	**Model finetuning setup**	CLIN (n = 1340 patients)		PAT (n = 400 patients)	
		AI	Derm	AI	Derm
Baseline (on this subset)		72.6% (69.8, 76.9)	77.8% (75.3, 81.2)	69.7% (64.3, 76.8)	86.8% (82.7, 89.4)
Score recalibration	Same model	76.8% (71.8, 81.4)		77.8% (70.2, 84.1)	
Training distribution matched to Stanford	Model **retrained** w/o Stanford case images	78.1% (70.1, 80.2)		78.4% (70.2, 84.1)	
Random split (20%)	Model **retrained** w/Stanford case images	74.2% (69.9, 78.1)		77.1% (70.2, 83.0)	
Condition aware split (20%)		83.7% (78.2, 89.1)		80.2% (76.9, 87.5)	
Condition aware split (20%) + score recalibration		85.3% (78.8, 90.2)		83.1% (78.1, 88.2)	
Condition aware split (20%)	Classifier layer **Fine tuned** w/Stanford case images	84.8% (78.8, 89.6)		83.4% (77.9, 88.5)	
Condition aware split (20%) + score recalibration		85.9% (79.1, 91.1)		84.2% (79.1, 88.8)	

The 95% confidence intervals are shown in parentheses.

Finally, we noticed that following retraining, the post-FiLM embeddings do not change substantially (Supplementary Fig. S1). Therefore, we hypothesised that a computationally more efficient approach would be to freeze both the image and metadata encoders and fine-tune only the final classification layer. This enables easier AI model adaptation because it requires only computational resources and labelled data to train a single layer of the AI model. Relative to re-training this model, this method also improved top-3 accuracy for CLIN and PAT by ∼13% (Fig. 4c).

## Discussion

Many AI technologies have been developed to interpret skin conditions^2^ and potentially augment the clinical decision making process.^18^, ^19^, ^20^ However, most have been tested in experimental settings and information is lacking on how to improve AI generalisation to different clinical settings. We assessed the generalisation of a skin condition AI on a dataset not previously seen by the model. The AI generalised well to both clinician-taken and patient-taken photographs and its accuracy was not associated with demographic factors. Instead, case ambiguity and condition distribution differences relative to the development set were the factors most strongly associated with cases with poorer accuracy.

Our results suggest that an efficient way to adjust a dermatological AI model to a new clinical setting with different condition distribution is to (1) augment the existing training data with conditions that are unexpected or less frequent, (2) fine tune just the classifier layer, and (3) use a temperature-based score recalibration to adjust the model confidence to match the new expected distribution. This method requires relatively low computational resources as it does not require retraining of the image or metadata encoders. The comparability of fine tuning solely the classification layer vs. end-to-end fine tuning also bodes well for generalising models developed using frozen foundation models where the encoder is frozen and one or more layers are trained on top of the frozen model. In terms of augmentation, in the absence of labelled data from the target distribution, some of these improvements can be obtained using *a priori* projected information about the anticipated condition distribution. These could include anticipated seasonal changes or known geographical differences in disease distribution.

A first key finding is that the AI model performs equally well for both CLIN and PAT photographs. Teledermatology, where the patient either sends photographs asynchronously or consults with their care provider via video, is becoming increasingly popular.^21^^,^^22^ As a result, both PAT and CLIN images are relevant, and it is comforting that AI models may potentially be deployed without requiring separate models for each photograph setting. Corroborating this, the AI embedding does not cluster by photograph source, implying its learned representation of CLIN and PAT photographs are similar. In other words, the AI’s understanding of features of a rash on a patient’s skin from different photographers, devices, and settings are similar. We further show that dermatologists tend to perform better on the PAT dataset, and observe that differences are associated with case ambiguity. For ambiguous cases—i.e., those where the differential diagnosis has uncertainties based on the images and structured medical history alone—dermatologists tend to rely on other information and tests, such as additional examination and targeted questions or biopsies to narrow down the differential diagnosis.^23^ One study limitation is that we lack information about recommended next steps, such as biopsy or in-clinic follow-up. This information would enable better characterisation of the source of ambiguity. Similarly, our cohort covers over 22 clinics across the San Francisco Bay Area, and although descriptive statistics appear relatively representative of the area,^24^ validation on a geographically and ethnically more diverse population is warranted.

Our second key finding is that condition distribution shifts between the AI development set and the previously unseen dataset contribute to the generalisation gap. Traditionally, most AI algorithms are trained based on the assumption that both the training and testing data are identically and independently distributed.^25^ However, this assumption rarely holds in reality, and can lead to prediction accuracy deterioration^26^^,^^27^ as seen here. Accounting for condition distribution differences is important because skin diseases vary widely by time (including both seasonal and non-seasonal variations) and geographic location.^28^^,^^29^ Our proposed method of adjusting the training or prediction distribution to match the expected condition distribution and fine tuning the classifier can help mitigate AI performance regressions in these scenarios, although we acknowledge that predicting distribution changes over time is challenging and likely varies based on the type of condition. One limitation of the training adjustment method, however, is that it requires labelled data which may be costly or challenging to obtain in the setting of rapid shifts. Alternatives include the prediction distribution recalibration based on expected changes and targeted labelling based on data point mapping in the embedding space, which appear relatively invariant to distribution shifts. A possible drawback of using both past and expected distributional changes, is the risk that the distribution changes in unexpected ways; however this risk applies in the absence of such techniques and may need non-technical solutions such as a use mode that involves human intervention as needed. In the event that additional data is available to train a generative model (or there exists access to a generative model already pre-trained on larger datasets), synthetically generated data may also hold promise to improve model performance.^30^^,^^31^ Additionally, beyond images, the AI model also takes as input patient history information such as the duration that the concern has been present for and whether they have a history of melanoma, eczema, psoriasis.^3^ These inputs could be broadened, such as with risk factors for cutaneous infections, to help close the generalisation gap.

Subject to satisfactory AI model performance on a cohort representing the target patient population, there may be several possible use cases of an AI model to interpret images of skin conditions. We note that AI models should be validated carefully and specifically to each intended use case, whether assistive or autonomous. An example of an assistive AI tool is an interface that could help assist primary care physicians and nurse practitioners with understanding and managing dermatologic conditions,^32^ whereas an example of an autonomous “background” system is a case triaging workflow that enables the most urgent cases to be seen first.^33^

In sum, our study presents a set of techniques based on the amount of information and data available to fine tune dermatological AI models to condition distribution differences that can arise in different clinical settings.

## Contributors

AL, GEH, MAS, DW, PB, Yuan Liu, Yun Liu, JK, SL jointly planned the study and drafted the protocol. GEH, MAS, MP, NB, BF, RS, KN, AE performed data collection and de-identification. RVR, AL, RS, Yuan Liu, Yun Liu, JK, SL conducted analysis of model results. RVR, AL, AJ, BM, JF, TS, Yuan Liu, Yun Liu conducted or supervised technical AI modelling. PS, MAS, DW provided administrative and logistical support. VM, PB, JK, and SL provided clinical input. YM, GSC, KC, DRW, JK, SL provided strategic guidance. RVR, RS, Yuan Liu, Yun Liu, JK, SL drafted the manuscript with input from all authors. RVR and AJ had access to the AI model outputs and verified the data; GEH and the data collection team verified the raw data prior to transfer. All authors have read and approved the final manuscript.

## Data sharing statement

The data used to develop the model is not publicly available due to restrictions in the data sharing agreements. However, we have collected and made additional skin image data available from appropriately consented individuals at https://github.com/google-research-datasets/scin A simplified version of the model that was trained similarly but provides the model embeddings for a more common single-image input setup, is provided at https://github.com/Google-Health/imaging-research/tree/master/derm-foundation.

## Declaration of interests

RVR, AL, PS, DW, Rory Sayres, AJ, BM, JF, TS, YM, GSC, KC, DRW, PB, Yuan Liu, Yun Liu are either current or past employees of Google and own Alphabet stock. JK is a co-inventor of systems and methods for automated clinical image quality assessment [U.S. Patent Application No. 17/937,714]. JK is the chair, American Academy of Dermatology Health Information Technology committee; former chair of American Academy of Dermatology Augmented Intelligence Committee. KC discloses patents for interface for Patient-Provider Conversation and Auto-Generation of Note or Summary, as well as patent for generating structured text content using speech recognition. KC is a board member of Lewa and Country Sun. VM received a $25 k grant for an e-delphi consensus process for ethics and AI from Stanford Centre for Bioethics (McCoy Centre). VM discloses royalties from Elsevier Ltd for textbook authorship and editing. VM is a stakeholder in World Health Organisation working group for AI in Health (unpaid). GEH, MAS, Rachna Sahasrabudhe, MP, NB, BF, KN AND AE have nothing to declare.
