# Supplementary material for: Closing the AI generalisation gap by adjusting for dermatology condition distribution differences across clinical settings
Source: eBioMedicine. 2025 Jun 2;116:105766. doi: 10.1016/j.ebiom.2025.105766 (PMC12167068; doi:10.1016/j.ebiom.2025.105766)
Supplement: Supplementary Figs. S1–S3 and Tables S1–S8 [file mmc1.docx]

## **Supplementary Material**

**Supplementary Tables and Figures**

**Supplementary Table 1: Top-3 Accuracy stratified by case ambiguity as measured by inter-rater agreement.**

| Dermatologist panel agreement rate | **CLIN** | | | **PAT** | | |
| --- | --- | --- | --- | --- | --- | --- |
|  | **% of data**  **(n patients)** | **AI** | **Derm** | **% of data**  **(n patients)** | **AI** | **Derm** |
| Unanimous agreement  (“**easy**”) | 29.5%  (557) | 0.8156  [0.777, 0.849] | 0.915  [0.887, 0.937] | 35.1%  (206) | 0.7216  [0.649, 0.786] | 0.949  [0.9087, 0.975] |
| 2 of 3 agreed  (“**medium**”) | 29.5%  (470) | 0.7457  [0.703, 0.785] | 0.746  [0.702, 0.786] | 29.5%  (154) | 0.7568  [0.679, 0.823] | 0.873  [0.807, 0.923] |
| Complete disagreement  (“**hard**”) | 40.7%  (284) | 0.6299  [0.591, 0.668] | 0.616  [0.572, 0.659] | 35.3%  (65) | 0.6158  [0.539, 0.688] | 0.729  [0.645, 0.803] |

**Supplementary Table 2**: **Number of conditions predicted by the AI (“k”) distribution for CLIN and PAT.** There is no significant difference observed between CLIN and PAT.

|  | **CLIN**  **(n = 1,681 patients)** | **PAT**  **(n = 569 patients)** |
| --- | --- | --- |
| **Average k**  **[IQR]** | 6.187  [3, 7] | 6.233  [3, 7] |

**Supplementary Table 3: Top-3 Accuracy stratified by demographic factors.**

| **Variable** | **Category** | **CLIN** | | **PAT** | |
| --- | --- | --- | --- | --- | --- |
|  |  | **AI** | **Derm** | **AI** | **Derm** |
| Sex | Female | 0.663  [0.631, 0.693] | 0.767  [0.736, 0.796] | 0.673  [0.619, 0.724] | 0.875  [0.832, 0.911] |
|  | Male | 0.672  [0.636, 0.705] | 0.769345  [0.736, 0.801] | 0.677  [0.611, 0.739] | 0.851  [0.793, 0.898] |
| Age | < 30 | 0.765  [0.713, 0.811] | 0.803  [0.752, 0.847] | 0.682  [0.582, 0.774] | 0.893  [0.807, 0.946] |
|  | [30, 40) | 0.625  [0.576, 0.672] | 0.759  [0.712, 0.801] | 0.606  [0.505, 0.700] | 0.875  [0.805, 0.927] |
|  | [40, 50) | 0.663  [0.601, 0.721] | 0.799  [0.741, 0.849] | 0.606  [0.505, 0.700] | 0.888  [0.808, 0.943] |
|  | [50, 60) | 0.646  [0.581, 0.706] | 0.762  [0.698, 0.818] | 0.671  [0.556, 0.773] | 0.871  [0.770, 0.939] |
|  | >= 60 | 0.648  [0.601, 0.692] | 0.737  [0.690, 0.780] | 0.694  [0.604, 0.775] | 0.806  [0.714, 0.879] |
| eFST | I-II | 0.66755  [0.633, 0.701] | 0.745  [0.711, 0.778] | 0.660819  [0.585, 0.731] | 0.901  [0.841, 0.943] |
|  | III-IV | 0.656  [0.618, 0.692] | 0.777  [0.741, 0.810] | 0.667  [0.590, 0.737] | 0.836  [0.767, 0.891] |
|  | V-VI | 0.623  [0.490, 0.744] | 0.800  [0.670, 0.896] | 0.750  [0.349, 0.968] | 0.714  [0.290, 0.963] |
|  | Unknown | 0.708995  [0.639, 0.773] | 0.820988  [0.753, 0.877] | 0.692  [0.622, 0.756] | 0.869  [0.809, 0.915] |

##

**Supplementary Table 4: Descriptive statistics of demographic variables dataset used to evaluate the fine-tuned model.** Compared with Table 1, there is no significant difference in the distribution of demographic variables in the resampled dataset.

|  |  |  | |
| --- | --- | --- | --- |
|  |  | **CLIN** | **PAT** |
| **Total number of cases after resampling** | | 1,340 | 400 |
| **Sex**  **(n, %)** | Female | 777 (58.0%) | 236 (59%) |
|  | Male | 563 (42.0%) | 164 (41%) |
| **Age**  **(n, %)** | ≥ 60 | 335 (25.0%) | 112 (28.0%) |
|  | 50-59 | 241 (18.0%) | 68 (17.0%) |
|  | 40-49 | 146 (11.0%) | 84 (21.0%) |
|  | 30-39 | 268 (20.0%) | 88 (22.0%) |
|  | < 30 | 348 (26.0%) | 48 (12.0%) |
| **eFST**  **(n, % excluding unknown)** | Unk. | 150 | 100 |
|  | V/VI | 60 (5.0%) | 9 (3.0%) |
|  | III/IV | 562 (47.2%) | 153 (51.0%) |
|  | I/II | 641 (47.8%) | 138 (46.0%) |

**Supplementary Table 5: Top-3 Accuracy stratified by demographic factors before and after fine tuning the model.**

| **Variable** | **Category** | **CLIN** | | **PAT** | |
| --- | --- | --- | --- | --- | --- |
|  |  | **Before** | **After** | **Before** | **After** |
| Sex | Female | 0.663  [0.631, 0.693] | 0.787  [0.741, 0.796] | 0.673  [0.619, 0.724] | 0.857  [0.821, 0.911] |
|  | Male | 0.672  [0.636, 0.705] | 0.780  [0.742, 0.801] | 0.677  [0.611, 0.739] | 0.871  [0.789, 0.898] |
| Age | < 30 | 0.765  [0.713, 0.811] | 0.813  [0.732, 0.847] | 0.682  [0.582, 0.774] | 0.839  [0.817, 0.946] |
|  | [30, 40) | 0.625  [0.576, 0.672] | 0.779  [0.722, 0.801] | 0.606  [0.505, 0.700] | 0.851  [0.815, 0.927] |
|  | [40, 50) | 0.663  [0.601, 0.721] | 0.799  [0.761, 0.849] | 0.606  [0.505, 0.700] | 0.899  [0.808, 0.943] |
|  | [50, 60) | 0.646  [0.581, 0.706] | 0.778  [0.687, 0.818] | 0.671  [0.556, 0.773] | 0.877  [0.780, 0.939] |
|  | >= 60 | 0.648  [0.601, 0.692] | 0.747  [0.691, 0.780] | 0.694  [0.604, 0.775] | 0.816  [0.714, 0.879] |
| eFST | I-II | 0.66755  [0.633, 0.701] | 0.755  [0.701, 0.778] | 0.660819  [0.585, 0.731] | 0.911  [0.841, 0.943] |
|  | III-IV | 0.656  [0.618, 0.692] | 0.777  [0.711, 0.810] | 0.667  [0.590, 0.737] | 0.863  [0.767, 0.891] |
|  | V-VI | 0.623  [0.490, 0.744] | 0.810  [0.671, 0.896] | 0.750  [0.349, 0.968] | 0.741  [0.390, 0.963] |
|  | Unknown | 0.708995  [0.639, 0.773] | 0.821  [0.754, 0.877] | 0.692  [0.622, 0.756] | 0.888  [0.819, 0.915] |

**Supplementary Table 6: Categorisation of skin conditions.**

| **Category** | **Conditions** |
| --- | --- |
| Contact dermatitis | Allergic Contact Dermatitis, Irritant Contact Dermatitis |
| Cutaneous Infections | Impetigo, Abscess, Cellulitis, Syphilis, Ecthyma, Erythrasma, Pitted keratolysis, Tinea, Deep fungal infection, Herpes Zoster, Herpes Simplex, Molluscum Contagiosum, Hand foot and mouth disease, Chicken pox exanthem, Insect Bite, Tinea Versicolor, Scabies, Paronychia, Candida, Skin and soft tissue atypical mycobacterial infection |
| Inflammatory Eruptions | Eczema, Psoriasis, Acne, Rosacea, Perioral Dermatitis, Intertrigo, Hidradenitis, Perleche, Folliculitis, Lichen planus/lichenoid eruption, Seborrheic Dermatitis, Chilblain, Pigmented purpuric eruption, Granuloma annulare, Infected eczema, Lichen nitidus, Lichen sclerosus, Erosive pustular dermatosis, Cutaneous sarcoidosis, Granuloma faciale, Granulomatous cheilitis. |
| Other Eruptions | Drug Rash, Acute generalised exanthematous pustulosis, Cutaneous lupus, Morphea/Scleroderma, Dermatitis herpetiformis, Dermatomyositis, Pityriasis rosea, Viral Exanthem, Erythema annulare centrifugum, Erythema multiforme, Erythema ab igne, Urticaria, Stasis Dermatitis, Photodermatitis, Hypersensitivity, Amyloidosis of skin, Keratosis pilaris, Prurigo nodularis, Inflicted skin lesions, Grover's disease, Confluent and reticulate papillomatosis, Keratoderma, Erythema nodosum, Zoon's balanitis, Xerosis, Lipodermatosclerosis, Retention hyperkeratosis, Lichenoid myxedema, Lymphomatoid papulosis, Skin striae, Sweet syndrome, Lichen striatus, Pruritic urticarial papules and plaques of pregnancy, Mastocytosis, Foreign body reaction of the skin, Fox-Fordyce disease, Pityriasis lichenoides, Miliaria. |
| Neoplasms | SK/ISK, Melanocytic Nevus, Cyst, Verruca vulgaris, Lentigo, Dermatofibroma, Atypical Nevus, Scar Condition, Milia, Becker's nevus, Epidermal nevus, Nevus sebaceous, Skin Tag, Adnexal neoplasm, Pearly penile papules. Pilonidal cyst, Cutaneous neurofibroma, Comedone, Angiokeratoma of skin, Clavus, Porokeratosis, Fordyce spots, Benign neoplasm of nail apparatus, Angiofibroma, Accessory nipple, Glomus tumour of skin, Lichen Simplex Chronicus, Pyogenic granuloma, Chondrodermatitis nodularis, Knuckle pads, Actinic Keratosis, SCC/SCCIS, Basal Cell Carcinoma, Melanoma, Cutaneous metastasis, Cutaneous T Cell Lymphoma, Condyloma acuminatum |
| Blisters and Ulcers | Canker sore, Pyoderma Gangrenosum, Venous Stasis, Ulcer, Bullous Pemphigoid, Pemphigus vulgaris, Bullosis diabeticorum, SJS/TEN, Traumatic bulla |
| Nail disorders | Onychomycosis, Longitudinal melanonychia, Onychorrhexis, Onycholysis, Beau's lines  Onychopapilloma, Nail dystrophy due to trauma, Trachyonychia, Onychomadesis, Leukonychia, Onychocryptosis |
| Hair Disorders | Androgenetic Alopecia, Alopecia Areata. Telogen effluvium, Hirsutism, Lichen planopilaris, Dissecting cellulitis of scalp |
| Pigmentary Disorders | Acanthosis nigricans, Post-Inflammatory hyperpigmentation, Vitiligo, Melasma, Post-Inflammatory hypopigmentation, Pityriasis alba, Idiopathic guttate hypomelanosis, Erythema dyschromicum perstans, Hemosiderin pigmentation of skin |
| Vascular | Cutaneous capillary malformation, Hemangioma, Leukocytoclastic Vasculitis, Hematoma of skin, Livedo reticularis, Livedoid vasculopathy, Varicose veins of lower extremity |
| Others | Ecchymoses, Burn of skin, Erythromelalgia, Lymphedema, Ichthyosis, Flushing, Foreign body, Notalgia paresthetica |

**Supplementary Table 7: Association between demographic, clinical and image quality factors and accuracy for (A) the AI and (B) Derm.**

(A)

| Variable | | AI (Univariable) | | AI (Multivariable) | |
| --- | --- | --- | --- | --- | --- |
|  |  | Log Odds | P-value | Log Odds | P-value |
| Age | 60-90 | REF | | | |
|  | 50-60 | 0.14  [-0.23, 0.5] | 0.46 | 0.29  [-0.12, 0.69] | 0.17 |
|  | 40-50 | 0  [-0.34, 0.35] | 0.98 | -0.02  [-0.4, 0.37] | 0.94 |
|  | 30-40 | -0.06  [-0.36, 0.24] | 0.71 | 0.04  [-0.3, 0.38] | 0.81 |
|  | 0-30 | 0.46  [0.11, 0.81] | < 0.05 | 0.41  [0.02, 0.81] | < 0.05 |
| eFST | Unk | REF | | | |
|  | V/VI | -0.38  [-1, 0.25] | 0.24 | -0.48  [-1.18, 0.22] | 0.18 |
|  | III/IV | 0.03  [-0.26, 0.31] | 0.85 | 0.04  [-0.3, 0.38] | 0.82 |
|  | I/II | 0.06  [-0.22, 0.34] | 0.66 | 0.05  [-0.3, 0.4] | 0.79 |
| Sex | Female | REF | | | |
|  | Male | -0.07  [-0.29, 0.34] | 0.52 | -0.02  [-0.27, 0.22] | 0.85 |
| Inter-rater agreement | Ambiguous | REF | | | |
|  | Intermediate | 0.8  [0.51, 1.09] | <0.0001 | 0.81  [0.5, 1.12] | < 0.0001 |
|  | Unambiguous | 1.17  [0.91, 1.43] | <0.0001 | 1.14  [0.85, 1.42] | < 0.0001 |
| Condition Category | Others | REF | | | |
|  | Neoplasms/Growths | 1.76  [0.7, 2.82] | < 0.01 | 1.54  [0.42, 2.66] | <0.01 |
|  | Other Eruptions | 0.67  [-0.39, 1.74] | 0.22 | 0.5  [-0.62, 1.63] | 0.38 |
|  | Cutaneous Infections | 1.12  [0.05, 2.18] | < 0.05 | 0.8  [-0.32, 1.93] | 0.16 |
|  | Contact Dermatitis | 1.16  [0.1, 2.22] | < 0.05 | 0.97  [-0.14, 2.09] | 0.09 |
|  | Inflammatory Conditions | 2.71  [1.61, 3.81] | < 0.0001 | 2.61  [1.45, 3.76] | <0.0001 |
|  | Healthy | 0  [-4, 4] | 0.97 | 0  [-4, 4] | 0.88 |
|  | Neuropathic Disorders | 0  [-4, 4] | 0.93 | 0  [-4, 4] | 0.86 |
|  | Birthmarks | 0.81  [-1.26, 2.87] | 0.44 | -0.48  [-2.63, 1.67] | 0.66 |
|  | Blisters | -0.01  [-1.64, 1.63] | 0.99 | 0.02  [-1.68, 1.72] | 0.98 |
|  | Ulcers | -0.68  [-3.17, 1.8] | 0.59 | -1.21  [-3.76, 1.34] | 0.35 |
|  | Oral Disorders | 0  [-4, 4] | 0.97 | 0  [-4, 4] | 0.90 |
|  | Vascular | 0.86  [-0.4, 2.12] | 0.18 | 0.6  [-0.73, 1.94] | 0.37 |
|  | Pigmentary Disorders | 1.6  [0.33, 2.88] | < 0.05 | 1.42  [0.08, 2.75] | < 0.05 |
|  | Nail Disorders | 1.31  [0.05, 2.58] | < 0.05 | 1.15  [-0.17, 2.48] | 0.09 |
|  | Hair Disorders | 0  [0.54, 4] | < 0.05 | 2.01  [0.07, 3.96] | < 0.05 |
| Anatomic Location | Unspecified | REF | | | |
|  | Anterior Torso | 0.15  [-0.41, 0.71] | 0.59 | 0.17  [-0.44, 0.78] | 0.59 |
|  | Posterior Torso | 0.11  [-0.51, 0.73] | 0.72 | 0.11  [-0.58, 0.79] | 0.76 |
|  | Scalp | 0.49  [-0.4, 1.39] | 0.28 | 0.32  [-0.69, 1.32] | 0.54 |
|  | Mouth and Tongue | -0.52  [-1.72, 0.69] | 0.40 | -0.18  [-1.52, 1.16] | 0.79 |
|  | Leg | 0.33  [-0.21, 0.88] | 0.23 | 0.43  [-0.17, 1.03] | 0.16 |
|  | Head, Neck and Face | 0.14  [-0.38, 0.65] | 0.6 | -0.22  [-0.79, 0.36] | 0.47 |
|  | Hand and Palm | -0.16  [-0.78, 0.47] | 0.62 | -0.02  [-0.71, 0.68] | 0.96 |
|  | Genitalia | -0.62  [-1.29, 0.05] | 0.07 | -0.74  [-1.49, 0.01] | 0.05 |
|  | Dorsal Foot | -0.15  [-0.85, 0.56] | 0.68 | 0.09  [-0.69, 0.88] | 0.81 |
|  | Buttock | 0.36  [-0.65, 1.37] | 0.49 | 0.46  [-0.6, 1.53] | 0.40 |
|  | Arm | -0.21  [-0.74, 0.33] | 0.45 | -0.15  [-0.74, 0.45] | 0.63 |
| Photograph Source | CLIN | REF | | | |
|  | PAT | -0.19  [-0.38, 0.06] | 0.13 | -0.29  [-0.62, 0.04] | 0.09 |
| eConsult year | After 2020 | REF | | | |
|  | Before 2020 | -0.16  [-0.38, 0.06] | 0.16 | -0.09  [-0.41, 0.24] | 0.59 |
| Number of photos per case | Less than 3 | REF | | | |
|  | 3 or more | -0.01  [-0.23, 0.21] | 0.96 | 0.1  [-0.15, 0.35] | 0.45 |
| Has blurry images? | No | REF | | | |
|  | Yes | -0.09  [-0.42, 0.24] | 0.59 | 0.01  [-0.36, 0.38] | 0.96 |
| Has images with lighting issues? | No | REF | | | |
|  | Yes | -0.08  [-0.54, 0.38] | 0.72 | -0.05  [-0.57, 0.47] | 0.84 |
| Has images with non-skin regions? | No | REF | | | |
|  | Yes | -0.38  [-0.77, 0.01] | 0.06 | -0.59  [-1.04, -0.14] | < 0.05 |

(B)

| Variable | | Derm (Univariable) | | Derm (Multivariable) | |
| --- | --- | --- | --- | --- | --- |
|  |  | Log Odds | P-value | Log Odds | P-value |
| Age | 60-90 | REF | | | |
|  | 50-60 | 0.29  [-0.11, 0.7] | 0.16 | 0.46  [0.02, 0.91] | < 0.05 |
|  | 40-50 | 0.36  [-0.04, 0.76] | 0.08 | 0.42  [-0.02, 0.85] | 0.06 |
|  | 30-40 | 0.18  [-0.15, 0.51] | 0.29 | 0.28  [-0.1, 0.65] | 0.15 |
|  | 0-30 | 0.44  [0.06, 0.81] | **< 0.05** | 0.48  [0.05, 0.91] | **< 0.05** |
| eFST | Unk | REF | | | |
|  | V/VI | -0.26  [-1.03, 0.52] | 0.52 | -0.04  [-0.9, 0.81] | 0.92 |
|  | III/IV | -0.33  [-0.67, 0.01] | 0.06 | 0.02  [-0.38, 0.41] | 0.94 |
|  | I/II | -0.47  [-0.8, -0.14] | < 0.01 | -0.09  [-0.49, 0.32] | 0.67 |
| Sex | Female | REF | | | |
|  | Male | -0.01  [-0.25, 0.24] | 0.96 | -0.04  [-0.31, 0.24] | 0.80 |
| Inter-rater agreement | Ambiguous | REF | | | |
|  | Intermediate | 0.73  [0.43, 1.04] | < 0.0001 | 0.74  [0.42, 1.06] | < 0.0001 |
|  | Unambiguous | 1.75  [1.43, 2.07] | < 0.0001 | 1.73  [1.4, 2.07] | < 0.0001 |
| Condition Category | Others | REF | | | |
|  | Neoplasms/Growths | 1.42  [0.35, 2.49] | < 0.01 | 0.9  [-0.28, 2.07] | 0.14 |
|  | Other Eruptions | 0.8  [-0.29, 1.88] | 0.15 | 0.56  [-0.62, 1.75] | 0.35 |
|  | Cutaneous Infections | 1.33  [0.24, 2.42] | < 0.05 | 0.88  [-0.32, 2.07] | 0.15 |
|  | Contact Dermatitis | 0.91  [-0.15, 1.98] | 0.09 | 0.46  [-0.71, 1.63] | 0.44 |
|  | Inflammatory Conditions | 0.83  [-0.24, 1.89] | 0.13 | 0.33  [-0.84, 1.5] | 0.58 |
|  | Healthy | 0  [-4, 4] | 0.91 | 0  [-4, 4] | 0.90 |
|  | Neuropathic Disorders | 0  [-4, 4] | 0.91 | 0  [-4, 4] | 0.89 |
|  | Birthmarks | 0  [-4, 4] | 1.00 | 0  [-4, 4] | 0.92 |
|  | Blisters | -0.4  [-2.01, 1.21] | 0.63 | -0.24  [-1.95, 1.47] | 0.78 |
|  | Ulcers | 0  [-4, 4] | 1.00 | 0  [-4, 4] | 0.93 |
|  | Oral Disorders | 0  [-4, 4] | 0.91 | 0  [-4, 4] | 0.85 |
|  | Vascular | 0.06  [-1.2, 1.32] | 0.92 | -0.69  [-2.08, 0.7] | 0.33 |
|  | Pigmentary Disorders | 2.09  [0.52, 3.66] | < 0.01 | 1.33  [-0.33, 2.99] | 0.12 |
|  | Nail Disorders | 1.4  [0, 2.8] | 0.05 | 0.96  [-0.56, 2.47] | 0.22 |
|  | Hair Disorders | 0  [-4, 4] | 1.00 | 0  [-4, 4] | 0.97 |
| Anatomic Location | Unspecified | REF | | | |
|  | Anterior Torso | -0.45  [-1.18, 0.27] | 0.22 | -0.7  [-1.47, 0.07] | 0.07 |
|  | Posterior Torso | -0.66  [-1.43, 0.11] | 0.09 | -0.99  [-1.81, -0.17] | < 0.05 |
|  | Scalp | 0.59  [-0.74, 1.92] | 0.38 | -0.14 [-1.56, 1.28] | 0.85 |
|  | Mouth and Tongue | -0.79  [-2.23, 0.64] | 0.28 | -0.52  [-2.13, 1.09] | 0.53 |
|  | Leg | -0.68  [-1.37, 0.02] | 0.06 | -0.82  [-1.56, -0.07] | < 0.05 |
|  | Head, Neck and Face | -0.53  [-1.21, 0.15] | 0.13 | -0.82 [-1.56, -0.09] | < 0.05 |
|  | Hand and Palm | -0.82  [-1.6, -0.04] | < 0.05 | -0.8 [-1.63, 0.03] | 0.06 |
|  | Genitalia | -0.49  [-1.38, 0.4] | 0.28 | -0.47  [-1.42, 0.49] | 0.34 |
|  | Dorsal Foot | -0.55  [-1.44, 0.34] | 0.23 | -0.62  [-1.57, 0.34] | 0.21 |
|  | Buttock | -0.16  [-1.39, 1.07] | 0.80 | -0.32  [-1.61, 0.97] | 0.63 |
|  | Arm | -0.71  [-1.41, -0.01] | < 0.05 | -0.83  [-1.58, -0.08] | < 0.05 |
| Photograph Source | CLIN | REF | | | |
|  | PAT | 0.62  [0.03, 0.93] | < 0.001 | 0.39  [-0.01, 0.8] | 0.05 |
| eConsult year | After 2020 | REF | | | |
|  | Before 2020 | 0.51  [0.26, 0.76] | < 0.0001 | 0.43  [0.06, 0.79] | < 0.05 |
| Number of photos per case | Less than 3 | REF | | | |
|  | 3 or more | 0.02  [-0.23, 0.26] | 0.89 | -0.1  [-0.37, 0.18] | 0.48 |
| Has blurry images? | No | REF | | | |
|  | Yes | 0.05  [-0.33, 0.43] | 0.79 | 0.12  [-0.31, 0.54] | 0.59 |
| Has images with lighting issues? | No | REF | | | |
|  | Yes | -0.47  [-0.94, 0] | < 0.05 | -0.36  [-0.88, 0.16] | 0.18 |
| Has images with non-skin regions? | No | REF | | | |
|  | Yes | -0.09  [-0.55, 0.36] | 0.69 | -0.29  [-0.81, 0.23] | 0.28 |

**Supplementary Table 8: STARD checklist.**

| Section & Topic | No | Item | Reported on |
| --- | --- | --- | --- |
|  |  |  |  |
| **TITLE OR ABSTRACT** |  |  |  |
|  | **1** | Identification as a study of diagnostic accuracy using at least one measure of accuracy  (such as sensitivity, specificity, predictive values, or AUC) | Abstract |
| **ABSTRACT** |  |  |  |
|  | **2** | Structured summary of study design, methods, results, and conclusions  (for specific guidance, see STARD for Abstracts) | Abstract |
| **INTRODUCTION** |  |  |  |
|  | **3** | Scientific and clinical background, including the intended use and clinical role of the index test | Introduction (first paragraph) and discussion (for intended use) |
|  | **4** | Study objectives and hypotheses | Introduction, last paragraph |
| **METHODS** |  |  |  |
| *Study design* | **5** | Whether data collection was planned before the index test and reference standard  were performed (prospective study) or after (retrospective study) | Methods, first paragraph |
| *Participants* | **6** | Eligibility criteria | Methods, first paragraph |
|  | **7** | On what basis potentially eligible participants were identified  (such as symptoms, results from previous tests, inclusion in registry) | Methods, first paragraph |
|  | **8** | Where and when potentially eligible participants were identified (setting, location and dates) | Methods, first paragraph |
|  | **9** | Whether participants formed a consecutive, random or convenience series | Methods, first paragraph |
| *Test methods* | **10a** | Index test, in sufficient detail to allow replication | Methods, “Skin condition classification AI” and Liu et al. reference |
|  | **10b** | Reference standard, in sufficient detail to allow replication | Methods, “Reference diagnosis” and Supplement |
|  | **11** | Rationale for choosing the reference standard (if alternatives exist) | N/A - no alternatives for differential diagnosis |
|  | **12a** | Definition of and rationale for test positivity cut-offs or result categories  of the index test, distinguishing pre-specified from exploratory | N/A - AI model provides categorical condition predictions directly, not continuous |
|  | **12b** | Definition of and rationale for test positivity cut-offs or result categories  of the reference standard, distinguishing pre-specified from exploratory | N/A, see above |
|  | **13a** | Whether clinical information and reference standard results were available  to the performers/readers of the index test | N/A, retrospective analysis |
|  | **13b** | Whether clinical information and index test results were available  to the assessors of the reference standard | Methods, “Reference diagnosis” |
| *Analysis* | **14** | Methods for estimating or comparing measures of diagnostic accuracy | Methods, “Statistical analysis” |
|  | **15** | How indeterminate index test or reference standard results were handled | N/A |
|  | **16** | How missing data on the index test and reference standard were handled | N/A |
|  | **17** | Any analyses of variability in diagnostic accuracy, distinguishing pre-specified from exploratory | N/A |
|  | **18** | Intended sample size and how it was determined | Methods, first paragraph |
| **RESULTS** |  |  |  |
| *Participants* | **19** | Flow of participants, using a diagram | Figure 1A |
|  | **20** | Baseline demographic and clinical characteristics of participants | Results, first paragraph, and Supplementary Tables 1-2 |
|  | **21a** | Distribution of severity of disease in those with the target condition | N/A - no severity information available or analysed |
|  | **21b** | Distribution of alternative diagnoses in those without the target condition | N/A |
|  | **22** | Time interval and any clinical interventions between index test and reference standard | N/A, retrospective analysis |
| *Test results* | **23** | Cross tabulation of the index test results (or their distribution)  by the results of the reference standard | N/A, cross tabulation not feasible for a large set of conditions. |
|  | **24** | Estimates of diagnostic accuracy and their precision (such as 95% confidence intervals) | Table 1 |
|  | **25** | Any adverse events from performing the index test or the reference standard | N/A, retrospective analysis |
| **DISCUSSION** |  |  |  |
|  | **26** | Study limitations, including sources of potential bias, statistical uncertainty, and generalisability | Discussion, third paragraph onwards. |
|  | **27** | Implications for practice, including the intended use and clinical role of the index test | Discussion, second-last paragraph |
| **OTHER INFORMATION** |  |  |  |
|  | **28** | Registration number and name of registry | N/A, retrospective analysis |
|  | **29** | Where the full study protocol can be accessed | N/A, retrospective analysis |
|  | **30** | Sources of funding and other support; role of funders |  |

**Supplementary Figure 1: UMAP projections before and after retraining the entire model.** Points are coloured by condition category. Refer to Figure 1C for legend.

| **Original** | **With re-training** |
| --- | --- |
| **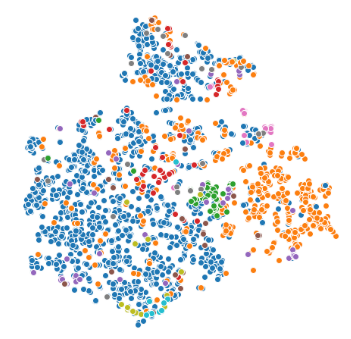** | **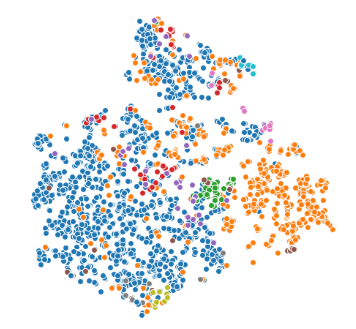** |

**Supplementary Figure 2: Examples of case images and respective saliency maps (computed via integrated gradients) before and after retraining.**

**a)**

| **Original** | **After re-training** |
| --- | --- |
| Healthy (0.337), Eczema (0.118), Tinea (0.093), Xerosis (0.093), Onychomycosis (0.052), Psoriasis (0.026), Onychocryptosis (0.022) | **Chillain (0.637)**, Healthy (0.118), Tinea (0.093), Xerosis (0.093), Onychomycosis (0.092), Onychocryptosis (0.022) |
| 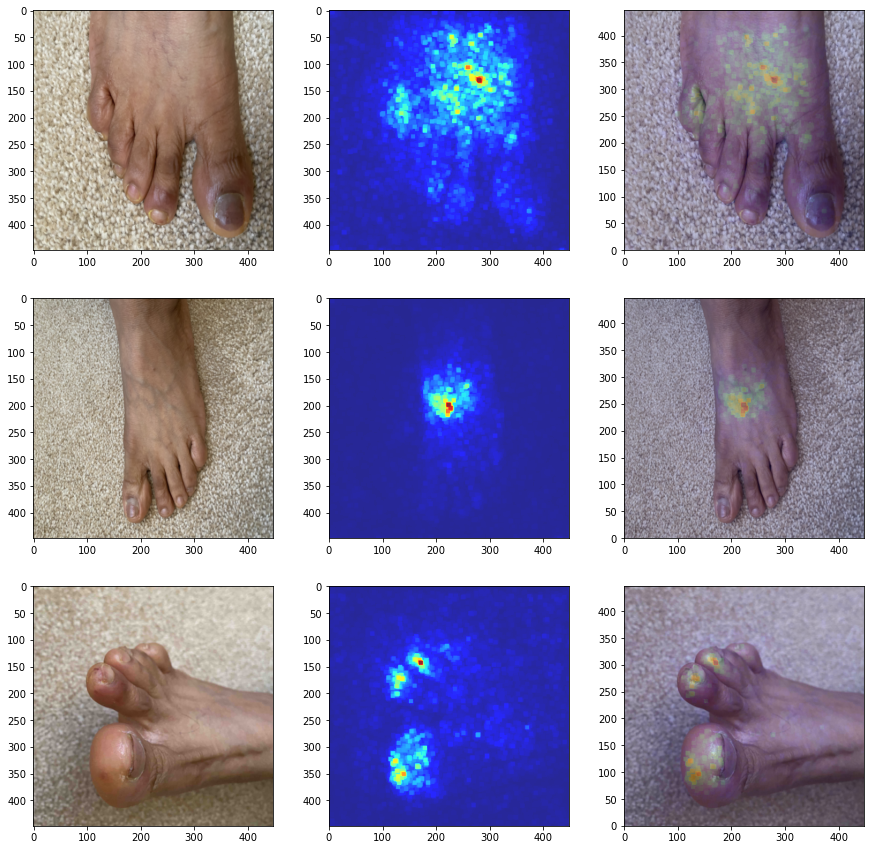 | 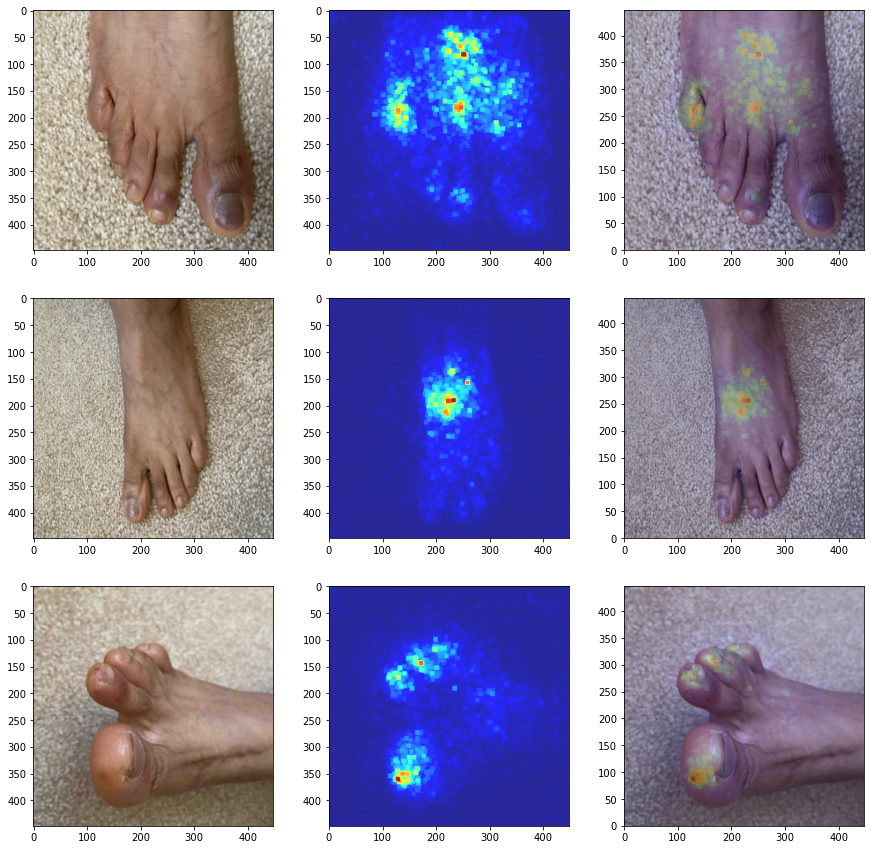 |

**b)**

| **Original** | **After re-training** |
| --- | --- |
| **Allergic Contact Dermatitis (0.149)**, Eczema (0.103), Tinea (0.073), Lichen planus/lichenoid eruption (0.054), Herpes Zoster (0.052), Granuloma annulare (0.050), Psoriasis (0.041) | **Allergic Contact Dermatitis (0.749)**, Eczema (0.103), Tinea (0.073), Lichen planus/lichenoid eruption (0.075) |
| 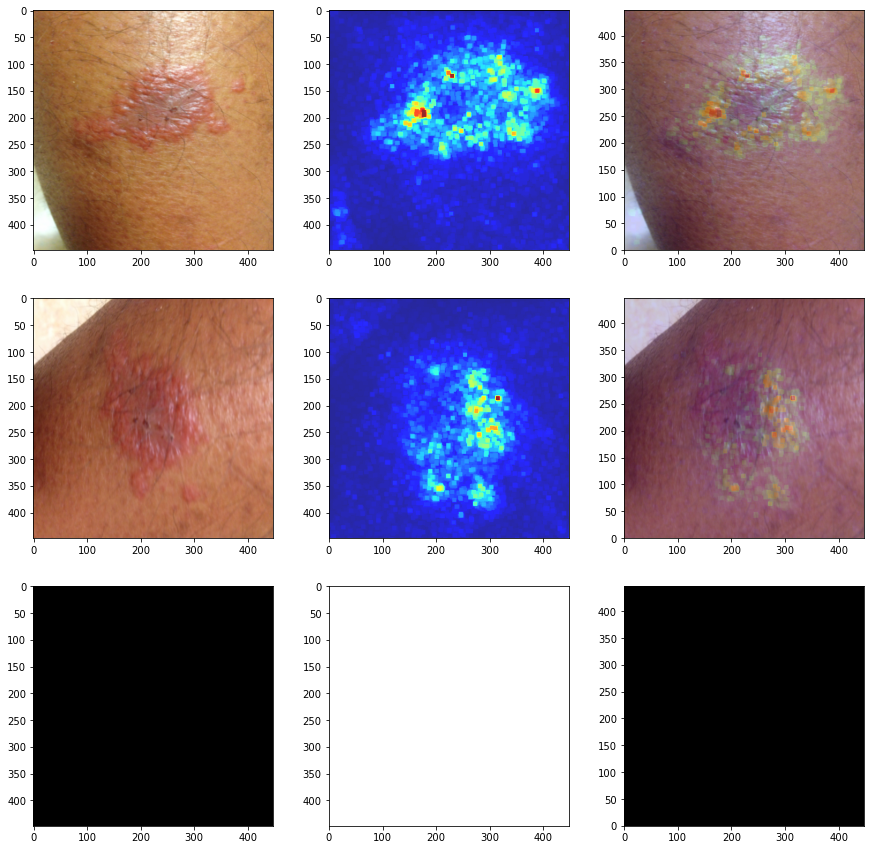 | 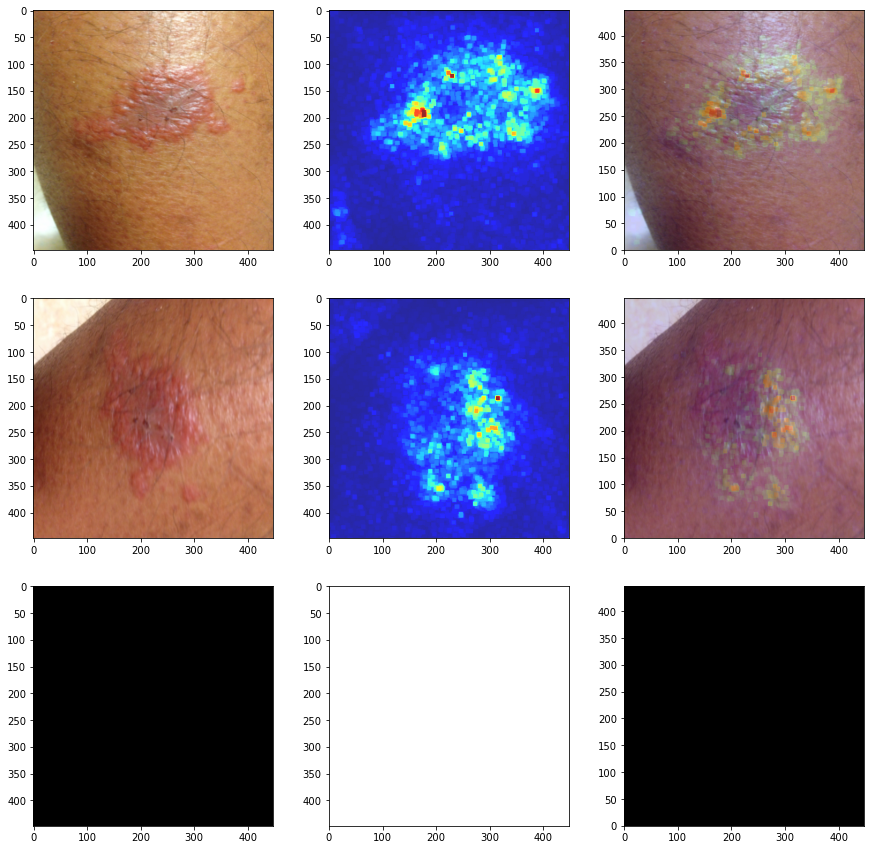 |

**Supplementary Figure 3: Visualisations similar to Figure 1c, but with 1 colour per category for visual clarity.**

**
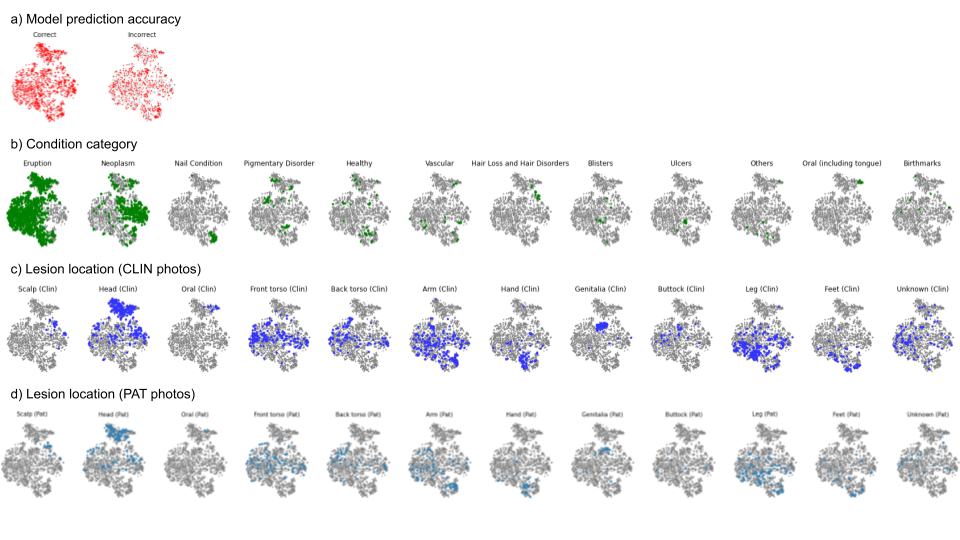
**
